# High-resolution imaging in studies of alcohol effect on prenatal development

**DOI:** 10.3389/adar.2023.10790

**Published:** 2023-02-01

**Authors:** Augustine Meombe Mbolle, Shiwani Thapa, Anna N. Bukiya, Huabei Jiang

**Affiliations:** 1 Department Medical Engineering, College of Engineering and Morsani College of Medicine, University of South Florida, Tampa, FL, United States,; 2 Department Pharmacology, Addiction Science and Toxicology, College of Medicine, The University of Tennessee Health Science Center, Memphis, TN, United States

**Keywords:** blood alcohol concentration, brain imaging, brain metabolism, maternal drinking, fetal development, alcohol *in utero*

## Abstract

Fetal alcohol syndrome represents the leading known preventable cause of mental retardation. FAS is on the most severe side of fetal alcohol spectrum disorders that stem from the deleterious effects of prenatal alcohol exposure. Affecting as many as 1 to 5 out of 100 children, FASD most often results in brain abnormalities that extend to structure, function, and cerebral hemodynamics. The present review provides an analysis of high-resolution imaging techniques that are used in animals and human subjects to characterize PAE-driven changes in the developing brain. Variants of magnetic resonance imaging such as magnetic resonance microscopy, magnetic resonance spectroscopy, diffusion tensor imaging, along with positron emission tomography, single-photon emission computed tomography, and photoacoustic imaging, are modalities that are used to study the influence of PAE on brain structure and function. This review briefly describes the aforementioned imaging modalities, the main findings that were obtained using each modality, and touches upon the advantages/disadvantages of each imaging approach.

## Introduction

Globally, alcohol (ethanol) is the most widely used psychotropic drug (1). Depending on gender and different countries, the drinking levels of alcohol can be considered light, moderate, heavy, or binge drinking. Moderate drinking involves one drink for women and two drinks for men in a day (2). Binge drinking can be typically classified as 4 or more drinks for women or 5 or more drinks for men consumed within a couple of hours of each other (3) leading to a blood alcohol concentration (BAC) level of 0.08 g/dL and higher (4). Moreover, heavy drinking can be reported as 8 or more drinks for women per week and 15 or more drinks for men per week (5).

According to the World Health Organization (WHO) Global Status Report on Alcohol and Health, in 2018, it was estimated that the total consumption of pure alcohol was 6.4 L per individual 15 years or older worldwide (6). In the United States, an estimated 38.5 million adults indulge in binge drinking per month, among which adults aged 18–34 years hold the highest prevalence (26%) (7). Excessive alcohol intake can lead to a plethora of detrimental effects targeting multiple organs such as the brain, liver, pancreas, and heart (8–12). Moreover, it increases the chance of developing various pathological conditions that include chronic diseases, cancers, and mental disorders (13–17). In particular, women of reproductive age are reported to be frequent users of alcohol (18, 19). Even more alarming, estimated global alcohol consumption rate during pregnancy is ~9.8% (20). The WHO European Region points at an average of 25.2% alcohol consumption rate during pregnancy. This statistic involves countries like Russia, United Kingdom, Denmark, Belarus, Ireland, Italy, France, and Finland (20). Whereas the WHO Eastern Mediterranean region (Oman, United Arab Emirates, Saudi Arabia, Qatar, Kuwait) reports the lowest average alcohol use at 0.2% among pregnant women (20). While socio-demographic (e.g., age, ethnicity, education level, reporting conditions, religious affiliation) and socio-economic (e.g., employment, nutritional diet, and prenatal care) factors play an essential role in the variability of alcohol consumption estimates (21–24), alcohol use among pregnant women does not decline. Between 2018 and 2020, the prevalence of alcohol consumption among pregnant women in the United States increased to 13.5%, and 5.2% were involved in binge drinking (25). Considering the deleterious effect of alcohol on health, alcohol use during pregnancy does not only affect pregnant women themselves but also their fetuses.

Although many women tend to stop or reduce drinking levels of alcohol once diagnosed with pregnancy, a high rate of unplanned pregnancies (45%) (26) may cause prenatal alcohol exposure (PAE) unknowingly during the first trimester. Collectively with the reported statistics on alcohol consumption prevalence during pregnancy, it can be inferred that a significant number of fetuses are exposed to the toxic effects of alcohol with or without the knowledge of women that they are pregnant. Such astonishing statistics inevitably leads to a plethora of health complications associated with PAE. In this review, we will briefly describe health concerns arising from alcohol exposure in utero, obstacles in their therapeutic treatment, and challenges faced by contemporary drug discovery efforts. We will then highlight the need for high-resolution imaging tools that would aid in the research process for assessment of pathophysiology and identification of promising drug targets for successful treatment of consequences arising from PAE. Finally, we will describe current advancements made in the field of high-resolution imaging that can be used as stepping stones for visualization of alcohol-related damage in small laboratory animals. We will conclude with the prospects of using high-resolution imaging at the cross-over of physics and biology for successful diagnostics and treatment of PAE-related health disorders.

## Health consequences of PAE

Alcohol is capable of easily and rapidly passing from the mother’s bloodstream via the placenta into the developing fetal circulation where it penetrates through blood-brain barrier, and targets multiple critical fetal organs (4, 27, 28). Alcohol can directly target several mechanisms at different stages of gestation and enable the teratogenic effects (29). These effects include disruption of neuronal cell survival, proliferation, and growth pathways leading to apoptosis (30) in the early gestation period, neonatal microglial abnormalities causing neuroinflammation (31), interference with the cortical vascular network development (32), alteration of cardiac progenitor cells gene expression (33), and dysfunction of the hypothalamus-pituitary-adrenal axis (34). Maternal alcohol consumption can result, first of all, in apparent gestational complications such as spontaneous miscarriage (35), premature delivery (36), low birth weight (37), placental abruption (38), first or second trimester bleeding, intraamniotic infection (39), and intrauterine growth restriction (40). Generally, higher BAC peaks of alcohol are associated with higher risks for adverse effects targeting physical, psychological, and behavioral development of the fetus (41, 42). Yet, based on a pregnancy cohort study from 8 metropolitan areas in the United States, it was found that every successive week of alcohol use led to an 8% increase in the risk of spontaneous abortion and did not correlate to the number of drinks consumed per week or to binge drinking (35). This underscores the significant fact that no known amount of alcohol is safe during pregnancy.

Fetal alcohol spectrum disorders (FASD) is the umbrella term that describes the detrimental effects of PAE and includes four distinct categories: fetal alcohol syndrome (FAS), partial fetal alcohol syndrome (pFAS), alcohol-related neurodevelopmental disorders (ARND) and alcohol-related birth defects (ARBD) (43). PAE causes lifelong consequences and allows FASD diagnosis mainly within four domains: the level of PAE, facial dysmorphology, growth deformities, and neurodevelopment retardation (44–48). However, not every neonate exposed to alcohol during gestation will develop FASD as it is estimated that only one in every 13 pregnant women exposed to alcohol would deliver a child with FASD (49). This could occur due to several factors such as the quantity, frequency, and timing of alcohol exposure, maternal age, diet, genetic and epigenetic factors along with the influence of other substance abuse (50–52). Yet, FASD are highly preventable neurodevelopmental disabilities with an estimated global prevalence of 0.77% which would result in 630,000 children born annually with FASDs worldwide (49). Unfortunately, the mechanisms causing FASDs are poorly understood, and no known cure has been developed (53).

FAS is the most severe form of FASD including craniofacial dysmorphic features, prenatal and postnatal fetal growth restriction, neurodevelopmental abnormalities, and cognitive or behavioral impairment (54). The three fundamental facial features of FAS include short palpebral fissures, smooth philtrum, and thin vermilion border of the upper lip; the cranial features include smaller head circumference, structural brain anomalies, and abnormal neurophysiology and in some cases recurrent non-febrile seizures (55). Various studies have demonstrated that PAE decreases the bioavailability of glutamine and glutamine-related amino acids and hence hinders fetal development (56, 57). It is reported that 0.15% of live births result in FAS globally and this percentage rises in countries that are characterized by a higher consumption of alcohol during pregnancy (e.g., Belarus, Italy, Ireland, Croatia, and South Africa) (20). However, in the case of pFAS, only a few characteristic features of FAS are present such as facial dysmorphology, neurocognitive impairment, and either growth restriction or microcephaly (44).

ARND is the most prevalent yet difficult form of FASD to be diagnosed (58). ARND includes neurocognitive and behavioral impairments but lacks the presence of distinct FAS cranial and facial phenotypes, consequently remaining undiagnosed or misdiagnosed (44, 54, 58). PAE induces neurotoxic effects resulting in morphological or functional alterations of specific neuronal structures and brain circuits (59, 60). In an observational cohort study, it was found that moderate or binge drinking during pregnancy disrupts the cortical connectivity and impairs cognitive functions in children (61). Compelling evidence from various brain imaging and animal studies shows that PAE hampers cognitive function in various areas such as learning, memory, attention, speech development, vision, adaptive skills, and motor skills (62–67). Behavioral deficits observed include hyperactivity, impulsivity, poor social skills, aggressive behavior, and mood disorders (62, 67, 68). A dose-dependent prenatal alcohol exposure study done by Lees et al. (2020) found evidence of differences in cerebral and regional brain volume associated with psychological and behavioral problems among adolescents aged 9–10 years (69). Neuroimaging studies also show youths exposed to heavy maternal alcohol exposure with smaller cerebral surface area and irregular cortical thickness in comparison to unexposed youths (70–72). Attention deficit hyperactivity disorder has high comorbidity with FASD and has been found to have a 48% prevalence among children diagnosed with FASD (73). ARNDs are often missed due to features that can overlap with several different neurodevelopmental disorders or can often be credited to environmental or socioeconomic factors for behavioral deficits.

ARBD fall under the rarer spectrum of FASD which requires a history of PAE coupled with a major systemic malformation (44). This malformation includes cardiac (atrial septal defects, aberrant great vessels), auditory (neurosensory hearing loss), skeletal (radioulnar synostosis, vertebral segmentation defects, scoliosis), and ophthalmic (optic nerve hypoplasia, retinal vascular anomalies) or renal defect (horseshoe kidneys) (54). Among all the global congestive birth defects, it is estimated that 5% of the total cases are contributed by PAE (74–76). Indirect toxicity from alcohol metabolites (e.g., acetaldehyde) and impaired placental nutrition supply also lead to PAE-induced organ damage (77, 78). Congestive heart defects occur from acute, early alcohol exposure during the first gestation trimester in humans (54, 73). In an avian model study, the early co-administration of glutathione along with ethyl alcohol (ethanol) increased the percentage of embryos with normal hearts from 40% to 79% *via* inhibiting the action of PAE on reducing global DNA methylation (79). Studies have also shown PAE-induced alterations in neonatal lung development such as decreased lung mass and delayed lung maturation (80), inhibition of alveolarization and vascular development (81), and formation of hypoplastic lungs (82). There are experimental studies that show PAE deteriorates renal functions involving renal acidification, potassium excretion, and renal tubular cell use (83–85).

Despite the economic and public health burden, there are several obstacles to the diagnosis and treatment of health defects arising from PAE. Although early detection and intervention of PAE play an essential role in the prophylaxis of FASD, the lack of valid reliable methods for noting maternal alcohol exposure is an ongoing challenge. Although there have been several non-invasive methods such as passive surveillance systems, clinical studies, and meta-analyses, these observations largely depend on maternal self-report. Such self-reports can lack accurate assessment due to recall bias, societal stigma, and inconsistent screening. However, ethanol biomarkers can also be used as an early PAE detection tool. The direct metabolites of alcohol such as fatty acid ethyl esters (FAEE) in neonatal hair and meconium (86–88) and ethyl sulfate in maternal urine (86, 89) are present as distinct biological biomarkers. There are also several indirect metabolites of ethanol such as ethyl glucuronide in neonatal meconium or maternal hair (90–92) and phosphatidyl ethanol in maternal blood (93), although these indirect markers are less specific and indicative of alcohol exposure (86, 94). Still, no biomarker has been validated as a specific and sensitive diagnostic marker for PAE-induced toxic effects (86, 95). Clearly, there is an urgent need for bench studies that are aimed at better understanding of PAE pathophysiology and at finding markers and cures of deleterious consequences posed by PAE.

## Laboratory animal models to study PAE

While studies in humans offer immediate translation into the wide-scale clinical practice, standardization of drinking patterns, doses, and timing within a large maternal population represent an impractical and ethically challenging task (96, 97). Human studies are also inconsistent due to variable factors like maternal age, diet, genetics, social status, and multi-substance use (51, 52). Animal models present an invaluable research tool to study the molecular mechanisms by which alcohol exposure hampers prenatal development. The use of various animal species such as non-human primates, pig, sheep, and rodents allow for manipulating the drinking pattern, dose, timing, and control for other confounding factors. However, each species has advantages and disadvantages for studies focusing on PAE. For example, non-human primates closely match the gestational period of humans in terms of neurodevelopment and allow fetal magnetic resonance imaging (MRI) to assess PAE effects (98). Nevertheless, non-human primates are expensive models that are scarcely available and involve longer gestation periods and singleton pregnancies. Ovine species are also used for preclinical studies of FASD due to equivalent fetal brain size and body weight to a human fetus and comparable gestational period (147 days) (99). However, ovine models are characterized by ruminal fermentation and differ from the human metabolic pattern following alcohol ingestion (1, 100). Large animals like pigs produce large litters, express voluntary alcohol consumption and similar rates of alcohol intoxication and excretion as humans (101). Yet they lack the advantage of introducing genetic manipulations which are widely available in small rodents. The latter are the most widely used versatile research models that allow invasive molecular mechanism studies of fetal alcohol exposure. Rats are commonly used for FASD studies and demonstrate the structural, developmental, and behavioral deficits as in humans (102–104). Rats are also preferred over mice for behavioral studies as they are calmer, more social, and easier to examine learning and executive function (105, 106). Mouse models are smaller in size, easier to maintain, have a shorter gestation period, and larger offspring production. With the use of modern technology, mice offer genetic modeling and are available as transgenic, knock-in, and knock-out strains. Another advantage of mouse models is the development of similar dysmorphic features of FASD as observed in humans. Various studies show these observations including craniofacial dysmorphology (107), brain abnormalities (108), growth restriction (109), and cognitive deficits (110, 111). The disadvantage of using rodent models is the difference in gestation length where the third-trimester fetal development in humans is analogous to the early postnatal period of rodents (112). As a significant amount of brain development occurs postnatally among rodents (113), many studies administer ethanol to neonate pups, but the mechanisms of absorption, metabolism and excretion are significantly varied in prenatal and postnatal periods (114, 115). However, the major disadvantage of mouse model is the small fetal size that makes non-invasive imaging studies of brain development and its alterations by PAE barely feasible. Overcoming this limitation is paramount for further advancement of the field as current understanding of the neurobiology and pathophysiology of PAE and its teratogenic effects has been rooted in neuroimaging technologies, which have allowed researchers to study structural, metabolic, and physiological abnormalities resulting from PAE.

## High-resolution imaging techniques: Principles and major findings relevant to the field of PAE

High resolution imaging technologies could broadly be classified into structural neuroimaging technologies which identify neuroanatomical changes associated with PAE; functional neuroimaging technologies, which measure various neurophysiological signal changes associated with functional activities within various organs; and metabolic imaging modalities which detect various neurochemical changes by measuring the concentration of neurometabolites such as choline-containing compounds - which are markers of cell membrane stability and myelination, N-acetyl-aspartate (NAA)- which are markers of neuronal/axonal density and viability, and creatine/phosphocreatine, a marker of metabolic activities (116, 117) (Table 1). PAE mostly impacts the brain due to alcohol-related neurobiological damage in early development (118, 119). Thus, the brain is the most widely studied organ for the effects of PAE.

For the purpose of this review, we conducted a search in Google Scholar, PubMed, ScienceDirect and Web of Science for relevant literature using a combination of the following words: “prenatal alcohol exposure,” “neuroimaging,” “fetal alcohol spectrum disorder,” “FASD,” “fetal alcohol syndrome,” “magnetic resonance imaging,” “MRI,” “magnetic resonance spectroscopy,” “MRS,” “magnetic resonance microscopy,” “MRM,” “animal models,” “diffusion tensor imaging,” “DTI,” “functional MRI,” “fMRI,” “positron emission tomography,” “PET,” “single photon computed emission tomography,” “SPECT,” “photoacoustic tomography,” “functional.” Apart from the language, which was restricted to “English,” there were no restrictions in the date or subject of the study, and we examined each abstract to determine relevance of the literature. We further identified other studies by referring to the references of the studies obtained from the various databases. We ended up with a total of 71 articles for this review. Below, we describe the various neuroimaging modalities, in terms of their principles and major findings relevant to the field of PAE. We divide the modalities into three groups based on their use in studies of structural, functional, or metabolic effects of PAE. Table 2 summarizes the main finding of the various high-resolution imaging modalities in humans and animals.

### Structural neuroimaging technologies

#### Magnetic resonance imaging technologies (MRI)

MRI is a safe, non-invasive imaging modality capable of producing detailed three dimensional structural and functional information of tissues properties (120). MRI uses a strong magnet and radio frequency waves to measure tissue property-dependent signals from protons (water) within the living organisms. Tissue properties like density, local environment, blood oxygenation, water movement as well as relaxation properties (T1, T2) may influence the signal detectable by MRI in various ways. When irradiated with radiofrequency energy, protons within the tissue are forced to swing out of equilibrium with the MRI field becoming misaligned with it due to their spin. When the radiofrequency energy is turned off, the protons quickly realign with the field, releasing electromagnetic energy in the process. The electromagnetic energy (signal) detected and the time it takes the protons to realign with the magnetic field (T1, T2) are used to generate images of the tissue. Advancements in technology has led to the development of custom coils and more powerful magnets, capable of generating magnetic fields of up to 7.0 T and higher (107, 121). Various dyes and nanoparticles have also been developed for use in imaging contrast enhancement, resulting in high resolution MRI referred to as Magnetic Resonance Microscopy (MRM) (122–124). Unlike routine structural MRI, the resolution in MRM is in the micron scale, typically less than 100 microns. Modern systems now support about 21–43 microns isotropic resolution, with scanning time in the order of 30–120 min per specimen (107, 121). The diameter of the bore of the magnet is only about 5 cm, thus limiting the size of the imaged specimen. As a result, MRM is typically used in studies involving small animals like rodent models of PAE (107, 121, 125). It allows for imaging of embryos, as young as 10.5 days postfertilization (123), with the ability to view images in all planes simultaneously for morphological assessment.

**FIGURE 1:**
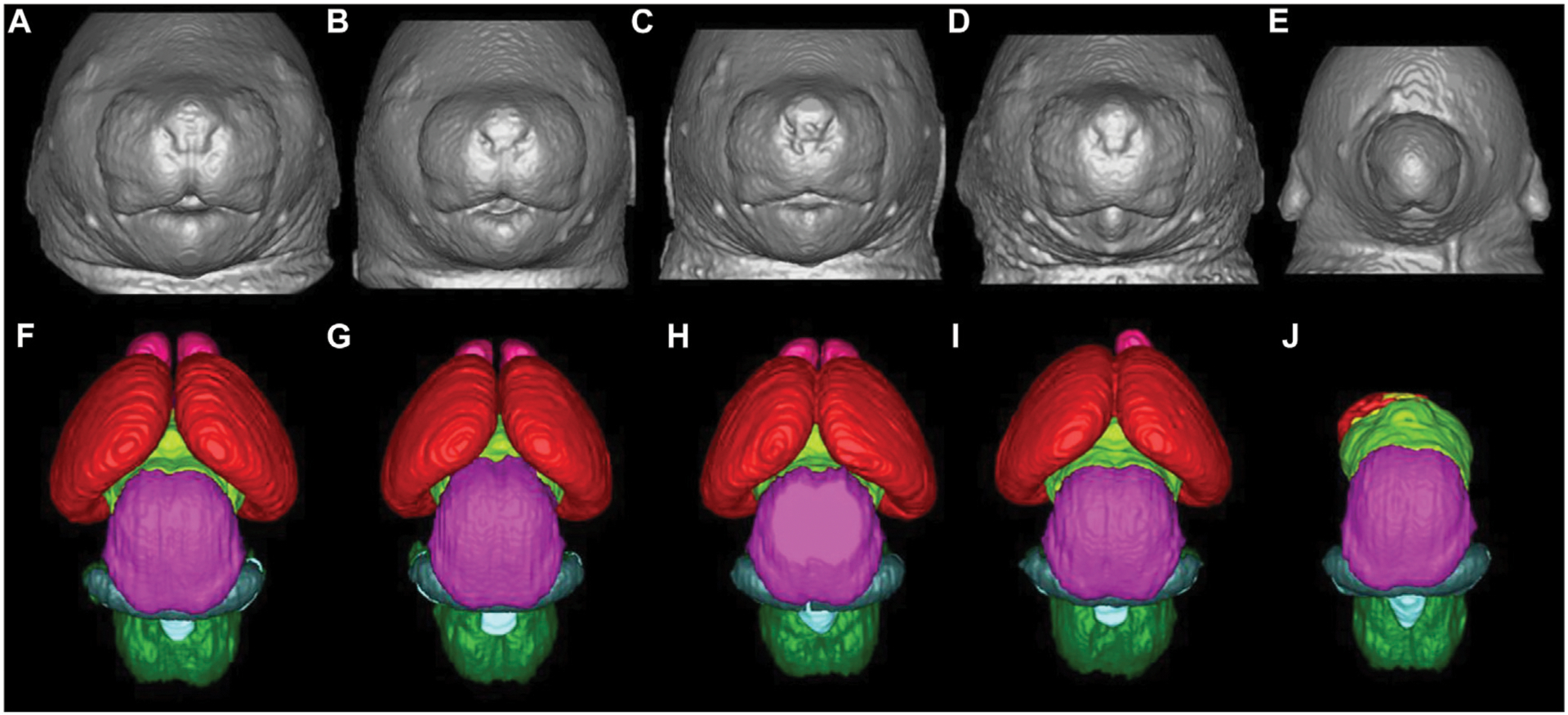
Facial and brain abnormalities following PAE on GD7 of the mouse. Compared to the control **(A)**, PAE-affected animals **(B-E)** show varying degrees of facial dysmorphology characterized by an elongated upper lip, a diminished philtra region, closely spaced nostrils, with small mandibles. The lower figures **(F-J)** show MRM-based 3D reconstructed brain anomalies from least to most severe. There is a correlation between facial dysmorphology and brain anomalies as animals with most subtle facial dysmorphology appear to have relatively normal brains. Animals with the more pronounced facial dysmorphology have a more severely affected brain, with the malformation corresponding to holoprosencephaly. **(E, J)**, is the most severe case with the brain completely missing most of its telencephalon with a severe facial phenotype, one nostril, with no lower jaw. Brain are color-coded as follows: olfactory bulbs (pink), cerebral cortex (red), diencephalon (lime green), midbrain (magenta), cerebellum (blue), mesencephalic/4th ventricle (teal), hindbrain (green). [Adapted from Ref. (11)].

Sulik et al. (107, 121, 126, 127) have characterized the developmental stage-dependent effects of PAE in mice using MRM-based analyses of fetal and postnatal mice. Timed C57B1/5J pregnant dams received a vehicle (control group) or two daily doses of intraperitoneal injection of 2.8–2.9 g/kg ethanol (ethanol group), administered at 4 h intervals on gestational days (GD) 7 and 8. Previous studies have shown that ethanol exposure on GD7 when early gastrulation occurs in mouse embryos, leads to a spectrum of craniofacial dysmorphology consistent with FAS (176, 177, 107). Similarly, GD8 lies within the early neurulation stage, and ethanol exposure at this stage has been shown to cause structural brain abnormalities (127). Control and ethanol-administered mice were stage-matched and on GD17, MRM was conducted on the fetal mice at either 7.0 T or 9.4 T. The resulting 29 μm isotropic resolution images were reconstructed and later processed using ITK-SNAP, a 3D segmentation/visualization software (128). Linear and volumetric morphological analyses was conducted with 3D reconstructions of selected brain, head/face and body regions obtained, and compared between the control and ethanol-administered groups. According to the results, acute ethanol exposure on GD7 results in a spectrum of facial and central nervous system defects, the most severe of which includes holoprosencephaly. As shown in Figure 1, the facial abnormalities may range from a slightly narrowed nose (a closely approximated nostril) and a slightly diminished central notch to an extremely narrowed snout and complete absence of a nostril. Furthermore, compared to the control, the lower jaw is deformed and appears short and narrow (126).

Compared to the control group, fetuses affected by ethanol in a mild fashion have brains looking fairly normal, but with smaller olfactory bulbs and a narrower space between cerebral hemispheres. As the severity of the teratogenic effect increases, olfactory bulbs may disappear completely and the hemispheres become indistinguishable across the midline (107, 121, 126, 127). Other GD7 ethanol exposure-induced abnormalities include cleft palate, pituitary dysgenesis, aglossia, aqueductal stenosis and eye abnormalities ranging from slight microphthalmia to bilateral anophthalmia (107, 121, 127). GD8 deformities noted in MRM scans include optic nerve coloboma, choanal atresia, narrowing of the cerebral aqueduct and third ventricle enlargement (107, 121, 126, 127).

Using MRM scans, regional brain segmentation and subsequent characterization of region-specific alterations and volumetric changes have equally been reported. Key amongst these findings in GD7 exposure models include volume reduction in telencephalic structures accompanied by increased lateral ventricular volume, mostly in fetuses with evident holoprosencephaly (126). GD8 exposure causes a disproportionate reduction in the volume of the olfactory bulb, hippocampus, as well as cerebellum, with a disproportional increase in the septal region and pituitary volumes (127).

#### Diffusion tensor imaging (DTI)

**FIGURE 2:**
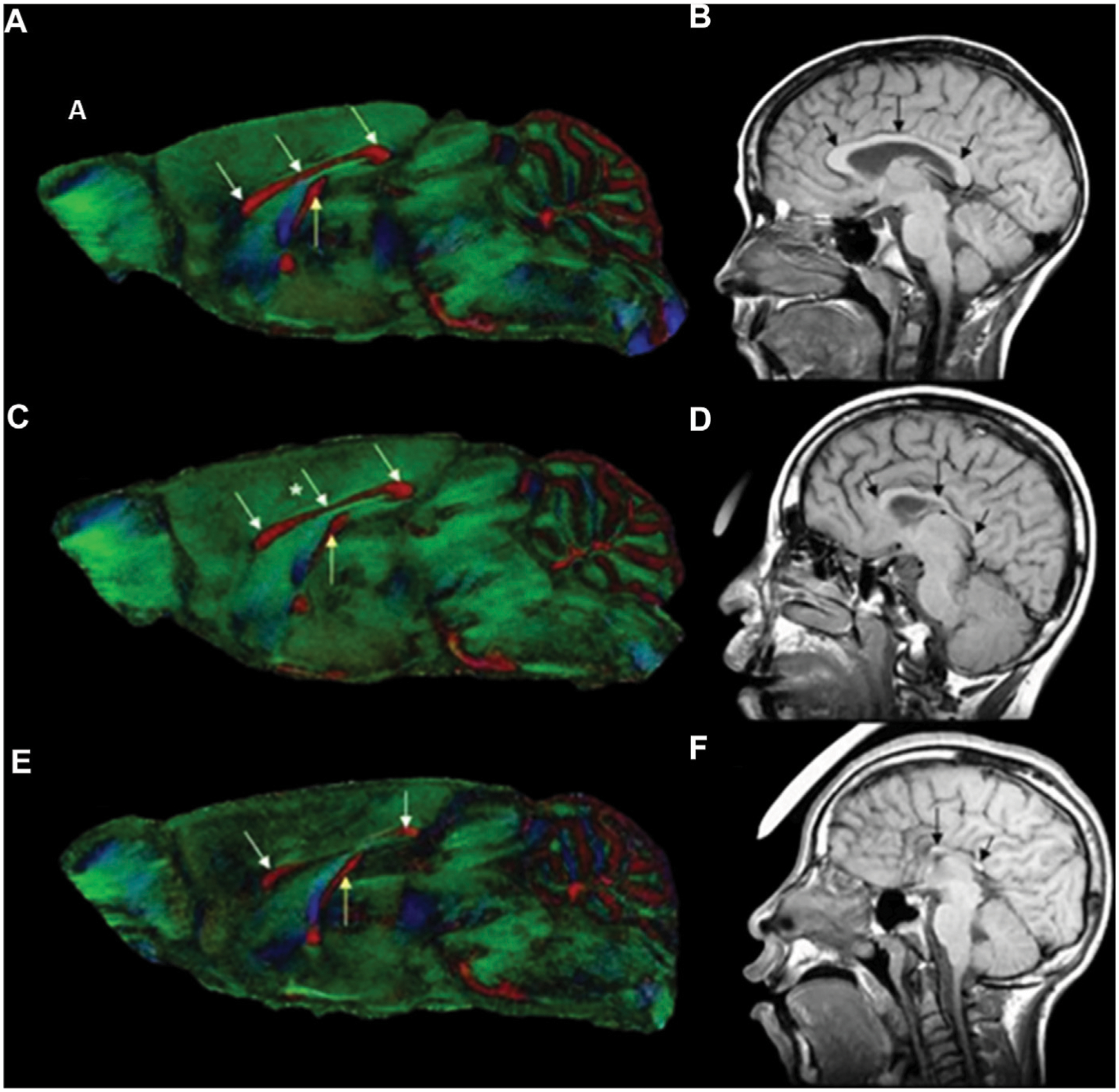
Color-coded fractional anisotropy maps from control mice **(A)** and GD7 ethanol exposed mice **(C, E)**, compared to a control individual **(B)** and FASD humans **(D, F)**. The ethanol exposed mice have varying degrees of brain dysmorphology compared to the control. The mouse in **(C)** has mild thinning of the corpus collosum in the middle section (*), while that in **(E)** has a reduced sized posterior and anterior corpus collosum with a completely absent middle part (see white arrows). The hippocampal commissure (yellow arrow) is also reduced in the more severely affected mice in **(E)**. The effect in mice is remarkably similar to that in humans with FASD. Compared to the control **(B)**, ethanol exposed humans **(D, F)** also have considerable dysmorphology of the corpus collosum (black arrows) [Adapted from (121)].

DTI is an emerging non-invasive MRI technology based on the measurement of the water molecule diffusions. The measured quantity is the diffusivity, a constant of proportionality that relates diffusive flux to concentration gradients (129). Due to the presence of numerous structures within tissue, the diffusion of water molecules is usually not isotropic. Thus, the measured diffusivity (diffusion tensor) is anisotropic, due to microscopic tissue heterogeneity (130). The diffusion tensor describes the diffusion of water molecules using a Gaussian model and results in a 3 × 3 symmetric positive-definite covariant matrix (131). The latter is capable of revealing the microstructural integrity of the white matter fiber tracts, enabling the quantification of subtle tissue changes affecting the integrity of the brain’s neural networks and interregional information transfer (132). White matter integrity is essential for effective functioning of a host of complex cognitive processes such as normal executive functions, attention, and processing speed (133–135). DTI measures the overall direction of diffusion of water molecules along white matter fiber tracts to access the structure and organization of different brain areas (136, 137). Two key scalar metrics are typically obtained from DTI. Firstly, fractional anisotropy, a scalar value between 0 and 1 which quantifies the overall directionality of diffusion and variation in axonal integrity. Secondly mean diffusivity, which describes the rotationally invariant magnitude of the average diffusivity and may primarily reflect myelin breakdown, changes in cellular density and volume. High fractional anisotropy and low mean diffusivity values are associate with healthier white matter microstructure whereas low fractional anisotropy and high mean diffusivity values are indicative of pathological white matter (70, 138). In the absence of discernable facial dysmorphology, such as in mild cases of PAE, high resolution DTI has proven to be effective in detecting ethanol-induced abnormalities in the white matter fiber tracts and has been applied in humans and animal studies alike. Specialized data analysis software such as DTI studio (139) and slicer3 (140) are used to create color-coded anisotropic maps from DTI data, to show the differing fiber orientation represented by the color-codes and the degree of diffusion anisotropy as represented by the signal intensity. DTI findings in human and animals (Figure 2) have revealed alterations in the corpus callosum, a structurally and functionally prominent brain commissural that actively connects the two cerebral hemispheres. These alterations (characterized by reduced fractional anisotropy and elevated mean diffusivity) range from complete agenesis of the corpus collosum to less severe alterations such as thinning and hypoplasia, with the thinning more localized in the posterior corpus collosum (141–145). Other quantitative studies have revealed disproportionate volume reduction in the genu and splenium of the corpus collosum of PAE subjects (146, 147). Sowell et al. (144, 145) identified dislocations in the posterior corpus collosum and correlated the degree of dislocation to the extent of facial dysmorphology.

#### Photoacoustic imaging for structural neuroimaging

**FIGURE 3:**
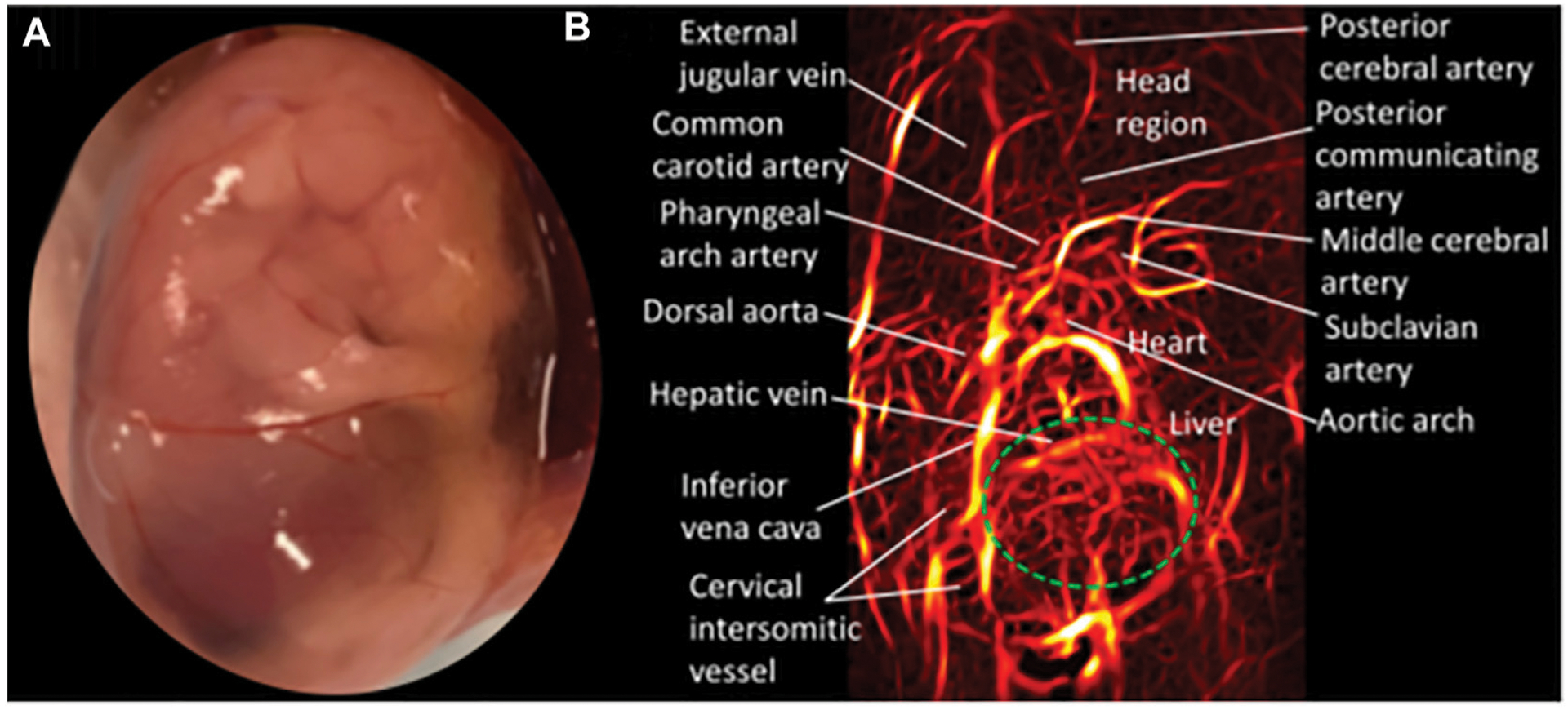
Structural photoacoustic tomography shows vascular tree of developing mouse embryoGD-17**. (A)** Photography of mouse embryo. **(B)** Photoacoustic image of fetal vasculature. Brain region within green oval shape [Adapted from (150)].

In photoacoustic imaging, laser light is used to generate ultrasound waves from tissue, by irradiating the tissue with typically nanoseconds pulsed laser light (148). The most used wavelengths for tissue excitation are the visible and near intra-red region, typically in the range 532–1,100 nm, with the near infrared region from 600–900 nm offering penetration depths extending to several centimeters. Once the tissue is irradiated with sufficient light energy of the right wavelength to cause optical excitation, specific tissue chromospheres namely hemoglobin, lipids, water, melanin, etc., absorb the light energy, which is then rapidly converted to heat energy by vibrational and collisional relaxation, producing a small temperature rise within the surrounding tissues (148, 149). The rise in temperature produced by the energy deposition, typically less than 0.1 K induces a thermoelastic expansion, accompanied by an initial pressure rise, which launches a pressure wave within the surrounding tissue. The pressure waves propagate to the tissue surface where they are detected by an acoustic transducer as a sequence of time-resolved electrical photoacoustic signals called A-lines. Jiang and colleagues (150) used structural photoacoustic tomography (sPAT) to study the effects of maternal ethanol consumption on fetal brain blood vessel diameter and density in second-semester equivalent (GD17) pregnant CD-1 mice models of PAE. (Figure 3).

Jiang et al. (150) used structural photoacoustic tomography (sPAT) to study the effects of maternal ethanol consumption on fetal brain blood vessel diameter and density in second-semester equivalent (GD17) pregnant CD-1 mice models of PAE. PAT images were acquired for 40 min (at 5 min intervals) following maternal intoxication of 20% ethanol at a volume of 3 g/kg *via* intraperitoneal injections. According to the results, maternal ethanol consumption on GD17 induces significant reduction in fetal brain vessel diameter (up to 31.25%) and vessel density (up to 25.1%)

### Functional neuroimaging technologies

Functional neuroimaging techniques measure the neurophysiological signal changes in various brain regions that result from PAE. Signal changes of interest are typically collected from the subject when no specific task is occurring (such as during sleep—“Resting state”), when subjects perform a given task or when subjects switch between tasks. These results provide information about the neuronal mechanisms underlying brain functions associated with sensory and cognitive activities.Functional magnetic resonance imaging (fMRI), single-photon emission computed tomography (SPECT), and positron emission tomography (PET) are amongst the functional imaging modalities reportedly used to study the effects of PAE. Emerging technologies such as functional multispectral photoacoustic imaging have also been used in recent studies.

#### Magnet-based imaging modalities

Functional magnetic resonance imaging (fMRI) is a specialized form of MRI commonly used to study brain functions. Established in the early nineties, fMRI employs the difference in magnetic susceptibility between oxygenated and deoxygenated hemoglobin and the changes in concentration that results from local neural activation; to measure blood oxygen level dependent magnetic resonance signals. Local neural activation results in a corresponding localized increase consumption of energy, resulting in differential blood oxygen levels and thus a different blood oxygen level dependent signal for oxyhemoglobin and deoxyhemoglobin. fMRI is the most widely used functional imaging modality for studying the effects of prenatal ethanol exposure.

**FIGURE 4:**
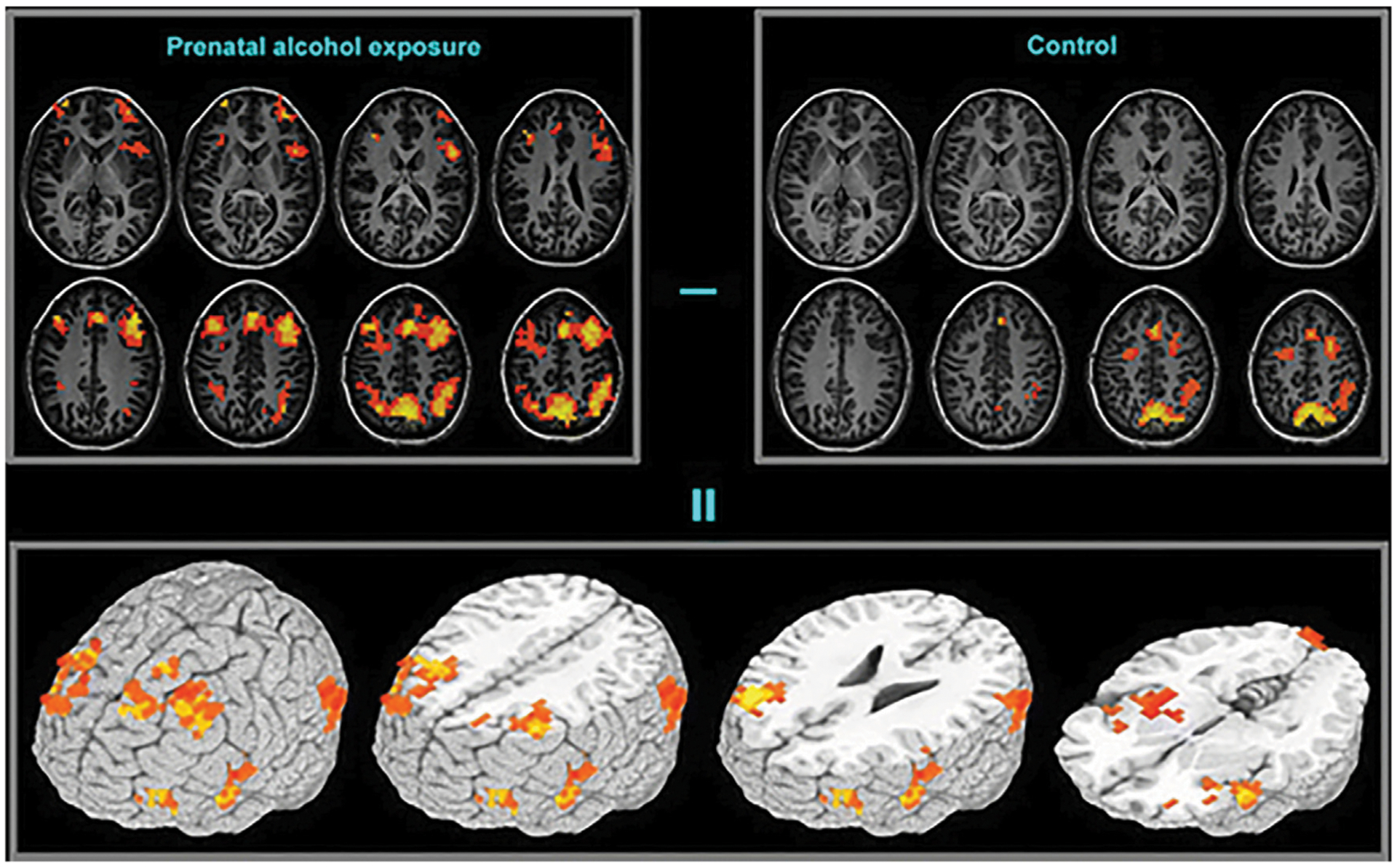
Functional brain activation differences (bottom frame) between the prenatal alcohol exposed (top-left frame) and control (top-right frame) subjects in a spatial working memory task. The exposed group exhibited greater activation in extended brain regions [Adapted from (151)].

fMRI studies have examined functional changes in brain activity relating to specific cognitive tasks in subjects of PAE, compared to normal control subjects (Figure 4). Hemodynamic responses in subjects exposed to ethanol prenatally have been studied during various cognitive tasks including response inhibition (152, 153), mathematics and number processing (46, 154) working memory (151, 155–158) and verbal learning (159). Most of these studies report a difference in activation in the frontal regions between FASD subjects and controls. In go/no-go tasks, greater neural activation has been observed in several frontal and parietal regions during response inhibition in PAE subjects (152).

#### Radiation-based imaging modalities

Single photon computed emission tomography (SPECT) is a non-invasive functional imaging modality that uses gamma radiations to evaluate blood flow or concentration of various neurotransmitters. A radioisotope is injected into the organ of interest and a gamma camera is used to capture 2D projections of the organ with the distribution of radiotracers from different angles. A computer algorithm is then used to reconstruct the 2D projections into a 3D image of the organ of interest. SPECT is typically used to evaluate regional brain metabolic activities by coupling blood flow to regional brain metabolic activities. SPECT studies have identified differences in cerebral blood perfusion in the temporal (161), parieto-occipital, and prefrontal lobes (161) of prenatal ethanol exposed subjects, differences in medial-frontal serotonin transporter binding and increased striatal dopamine transporter binding in prenatal ethanol exposed subjects (162).

Positron emission tomography (PET) is a non-invasive functional imaging modality that uses radiopharmaceutical isotopes called radiotracers to visualize and measure physiological activities. Radiation emitted from radiopharmaceuticals injected intravenously into a subject is registered by external detectors positioned at different orientations. The radiopharmaceutical injected into the organ of interest breaks down and emits positrons, which interact with free electrons resulting in an annihilation reaction (163). The two photons (gamma rays) emitted from the annihilation reaction travel in opposite directions and arrive coincidentally at 180° to each other at the external detector. This signal is transferred to a computer for processing. PET is typically used to quantitatively evaluate glucose metabolism and blood flow associated with brain activity.

SPECT/PET studies are somewhat limited in their use to study the effects of prenatal ethanol exposure possibly because they focus on the “resting brain” and thus do no provide a direct insight into specific behavioral deficits. A PET study using PET/fMRI (see below) has identified differences in regional cerebral metabolic rates in the thalamus and basal ganglion between prenatal ethanol exposed subjects and normal subjects (164).

#### Photoacoustic imaging for functional neuroimaging

Multispectral photoacoustic imaging is a form of optical absorption spectroscopy (165) which attempts to identify the source of photoacoustic imaging contrast by exciting tissue at multiple wavelengths and identifying various contrast sources by means of their known optical absorption spectra. The selected wavelengths are such that the different absorber can be distinguished from each other. After multi wavelength imaging, the resulting set of PAT images at each single wavelength are fed into a spectral unmixing algorithm, where they are converted to sets of images of specific absorbers.

**FIGURE 5:**
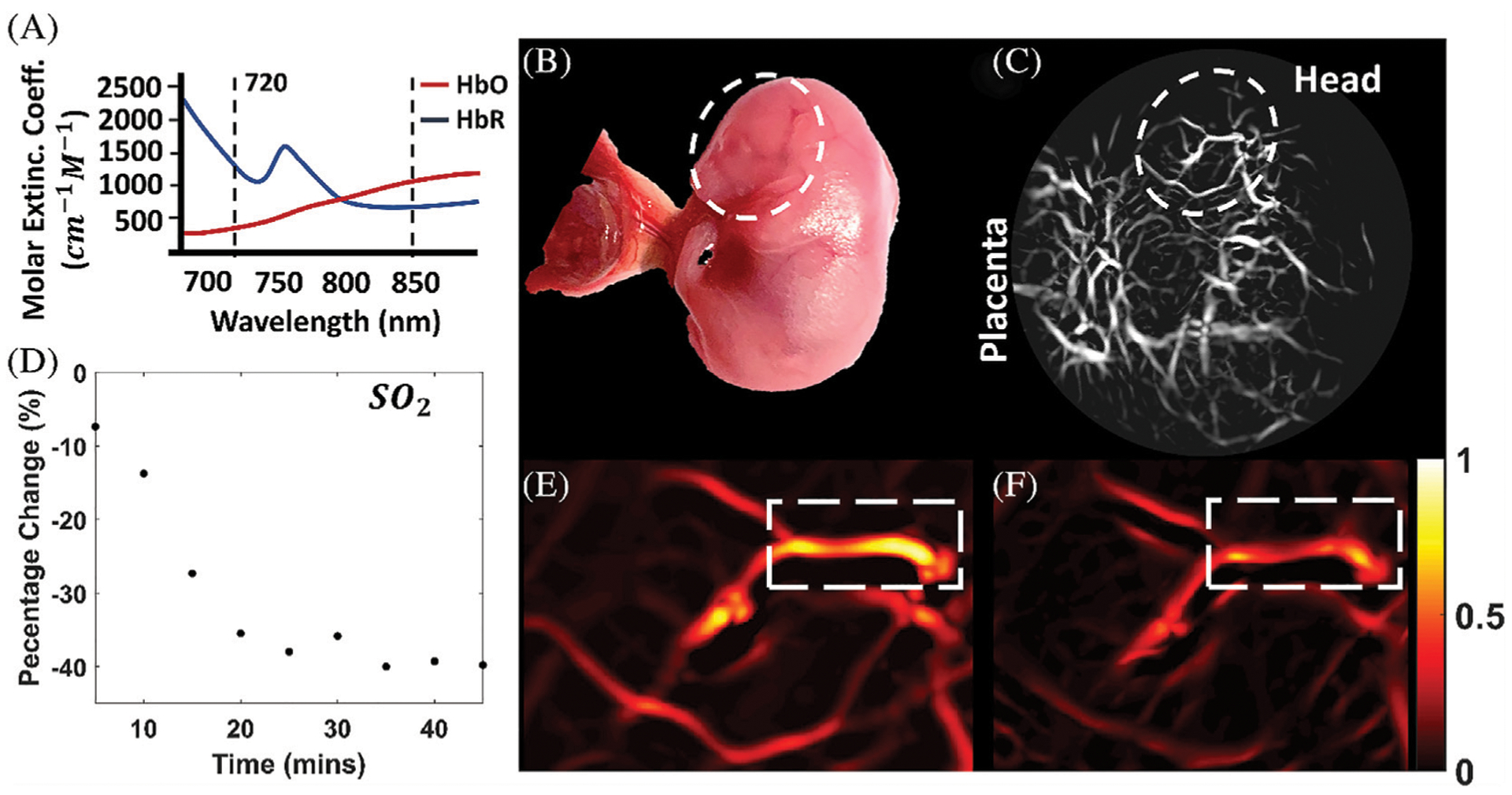
Multispectral photoacoustic imaging of oxygen saturation in fetal brain blood vessels following maternal ethanol intoxication on GD17. The multispectral data was acquired at the two wavelengths shown in **(A, B)**: Photograph of the mouse fetus**. (C)** Photoacoustic Images of the fetus**. (D)** Percentage change in oxygen saturation over time in selected blood vessels in **(E, F)**, oxygen saturation images [Adapted from (150)].

Jiang and colleagues (150) extended their photoacoustic imaging study of the effects of maternal ethanol consumption on fetal brain blood vessel in second-semester equivalent (GD17) pregnant CD-1 mice models of PAE by using multispectral photoacoustic tomography (fPAT) to study ethanol induced oxygen saturation on fetal brain blood vessels (Figure 5). Multispectral PAT images were acquired for 45 min (at 5 min intervals) following maternal intoxication of 20% ethanol at a volume of 3 g/kg via intraperitoneal injections. The results show that, maternal ethanol consumption on GD17 induces up to a 39.78% reduction in hemoglobin oxygen saturation in fetal brain blood vessels, indicative of significant hypoxia in fetal brain circulation.

### Modalities for imaging neurochemical (metabolic) effects of PAE

FASD studies in humans and animals typically use magnetic resonance spectroscopy (MRS) to study the metabolic effects of PAE. This involves studying changes in neurochemistry of various brain regions in PAE subjects and comparing the results to normal control subjects.

Magnetic resonance spectroscopy (MRS) is a non-invasive neuroimaging modality capable of providing biochemical information about specific brain regions (166, 167). When magnetic nuclei like ^1^H, ^31^P, ^13^C or ^19^F are placed in a magnetic field, they resonate at specific frequencies depending on the nuclei and the strength of the magnetic field. Thus, different radio frequency coils and hardware can be used to tune into these different frequencies to identify their origin. Due to the abundance in living tissue, and the strength of the magnetic resonant frequency, protons (^1^H) are by far the most widely used nuclei for MRS. Protons, contained in various biochemical molecules in living tissue resonate at different frequencies depending on the electronegativity of the chemical bond they are involved in. Based on these frequency differences [typically measured in parts per million (ppm) due to the small size] in biochemical molecules (metabolites) can be distinguished (168). An MRS experiment typically involves exciting the nuclei in a specific volume of tissue with a radiofrequency pulse and receiving the resulting signal, in the form of a spectrum of signal intensity versus frequency, over a range of frequencies. These spectra can then be analyzed to identify the chemicals present in the volume as well as their relative concentrations if the peaks are suitably calibrated (168). Typical brain neurochemicals quantified by MRS include N-acetyl aspartate (a neuronal integrity biomarker), choline (an essential molecule for the synthesis of the neurotransmitter acetylcholine and cell membrane constituent phosphatidylcholine), and creatine (an essential component for maintaining energy-dependent systems in cells, gamma-aminobutyric acid, glutamate and myoinositol) (166).

O’Leary et al. (170) used an animal model of neonatal ethanol exposure to study regional brain neurochemistry in developing rats. They administered ethanol to offspring early during postnatal life to mimic third trimester ethanol exposure in the human and used a specialized MRS technique called high-resolution magic angle spinning to ascertain and quantify neurochemical data from intact brain biopsies. The results from spectral analysis showed that neonatal ethanol exposure results in region specific alteration in a number of neurochemicals including glutamate, N-acetyl-aspartate, gamma-aminobutyric acids etc., with the most pronounced alterations occurring in the cerebellum. The findings are consistent with earlier results by Green et al. (178), who observed reduced levels of N-acetylaspartate and taurine (an inhibitory neuromodulator) in the cerebellum of both male and female neonatal rats following binge ethanol exposure; with lower glutamate levels in females, compared to controls. Several other studies employing proton (^1^H) MRS to study neurochemistry in PAE in humans and animals (46, 48, 168–170), have observed similar alterations in neurochemicals, with the most consistent results being a reduction in levels of neurochemicals like N-acetyl aspartate/creatine and N-acetyl aspartate/choline ratios in multiple brain regions, notably the parietal and frontal cortices, thalamus, and cerebellar dentate nucleus as well as the frontal white matter and corpus callosum (169).

## Conclusion and prospects

While epidemiology data on prevalence of PAE and resulting brain-targeted effects of FASD are staggering, high-resolution visualization of morphological and functional parameters of the brain lags behind. Variants of MRI technologies including MRM, DTI, and MRS, as well as radiation-based PET and SPECT imaging, are amongst the modalities consistently used to study the effects of PAE in humans and animals. FASD studies in humans and animals using various structural neuroimaging modalities have revealed several distinct abnormalities in the developing fetus owing to PAE. While some imaging modalities are specific to animals, others could be used in both animals and human subjects. Development of easily accessible high-resolution imaging approaches, such as photoacoustic imaging, holds promise for early diagnosis and successful therapeutic interventions in the field of PAE.

## Figures and Tables

**TABLE 1:** Classification of high-resolution imaging modalities based on functionality. Structural neuroimaging modalities are used to study neuroanatomical changes associated with PAE. Functional imaging modalities are used to study neurophysiological changes, specifically hemodynamic changes associated with PAE, while metabolic imaging modalities detect various neurochemical changes associated with PAE by measuring the concentration of neurometabolites.

Structural neuroimaging technologies	Functional neuroimaging technologies	Metabolic imaging technologies
Magnetic resonance microscopy (MRM) Diffusion tensor imaging (DTI) Structural magnetic resonance imaging Structural photoacoustic tomography (sPAT)	Functional magnetic resonance imaging (fMRI) Single-photon emission computed tomography (SPECT) Positron emission tomography (PET) Multispectral photoacoustic tomography (fPAT)	Magnetic resonance spectroscopy Single photon emission computed tomography (SPECT) Positron emission tomography (PET)

**TABLE 2:** High-resolution neuroimaging technologies used in the study of prenatal alcohol exposure.

Imaging modality (anatomical)	References/subject	Subject (age)	Findings
MRM	(126)	Mouse	Linear and volumetric analysis of MRM images of GD7 showed craniofacial dysmorphology and brain abnormalities, the most severe being holoprosencephaly (HEP), volumetric reduction in telencephalic structures, increased lateral ventricular volume in HEP
	(107)	Mouse	GD8 exposure results in optic nerve coloboma, choanal atresia, narrowing of cerebral aqueduct and 3rd ventricle enlargement
	(127)	Mouse	GD8 results in disproportionate reduction in olfactory bulb, hippocampus, cerebellum, along with a disproportionate increase in the sepal region and pituitary glands
	(172)	Mouse	GD-9 ethanol exposed mice presented with increase septal region width and a decreased cerebellar volume, along with enlargement of all ventricles. Noticeable misshapen cerebral cortex, hippocampus, and right striatum
	(171)	Mouse	GD7–11 ethanol exposed mice presented with significant decrease in cerebellar volume, along with increase septal volume GD12–16 ethanol treatment resulted in reduced hippocampal volume, along with enlarged pituitaries, and high incidence of edema/fetal hydrops
	(175)	Rat	GD1–20 exposed rats presented with reduced brain and isocortical volumes as well as isocortical surface area and thickness
DTI	(143)	Humans (Adult males 18 and over)	Alterations in the corpus callosum, ranging from thinning, hypoplasia, and complete agenesis. Reduced FA and elevated MD
	(146, 147)	Humans (children) (8–18 years old, mean age 13)	Disproportionate reduction in volume of genu and splenium
	(145)	Humans (Children) (7–11 years old, mean 13.8)	Dislocation in posterior corpus callosum, correlated to the extent of facial dysmorphology
	(173, 174)	Humans Children, aged 9.7–13.7)	Decreased FA in posterior portion of inferior longitudinal fasciculus and in left middle cerebellar peduncles (White matter)
	(175)	Rat	High FA in cerebral cortex
sPAT	(150)	Mouse	Maternal ethanol consumption on GD-17 induces significant reduction in fetal brain vessel diameter (up to 31.25%) and vessel density (up to 25.1%)
(Metabolic)			
MRS	(46, 48, 169, 170)	Monkey Rat	Reduction in levels of NAA/creatinine and NAA/Choline in multiple brain regions, notably parietal and frontal cortices, thalamus, cerebellar dentate nucleus, frontal white matter, and corpus callosum
(Functional)			
fPAT	(150)	Mouse	Maternal ethanol consumption on GD-17results in up to 39.78% reduction in hemoglobin oxygen saturation in fetal blood vessels, indicative of significant ethanol induced hypoxia in fetal brain circulation
fMRI	(152)	Humans (14.5 years old) Go/No-Go tasks	Similar Go/no-go task performance between groups. PAEs showed greater BOLD response across in prefrontal and cortical regions, but less response in caudate nucleus activation
	(151, 155, 156, 157)	Humans (Spatial working memory)	PAE children and adults showed overall less brain activity, but greater interior-middle frontal activity compared to controls during simpler activities
		Age matched Children (7–10) years old, Adults (18–33) years old	PAE showed greater BOLD response in frontal, insular, superior, middle, temporal, occipital, and subcortical regions
	(154)	Human adults (23.0 years old) (Arithmetic and number processing)	PAE exhibit lower accuracy but comparable reaction times, compared to controls
	(159)	Humans (10 years old) (Verbal working memory)	PAE showed increased activation in the left dorsal frontal, left interior parietal and bilateral posterior temporal regions
SPECT/PET	(164)	Human (20.6 vs. 22.8 years old) (Resting state)	Decreases in relative regional cerebral metabolic rates were found in 5 brain regions comprising thalamus and basal ganglia
	(161, 162)	Human (10.5 vs. 9.8-year old)	Significant brain volume reduction in PAEs
		Also (8.6 vs. 16 years old)	Reduced serotonin transporter binding in the medial frontal cortex and increased striatal dopamine transporter binding in PAEs
		(Resting state)	SPECT showed mild hypoperfusion of the left hemisphere (especially in parietooccipital and frontal regions) in PAEs
	(160)	Human (6–29 years old) and (29, 35 years old) (Resting state)	SPECT revealed at least 25% CBF reduction in the temporal region relative to the cerebellum

MRM, magnetic resonance microscopy; DTI, diffusion tensor imaging; MRS, magnetic resonance spectroscopy; sPAT, structural photoacoustic tomography; fPAT, functional photoacoustic tomography; fMRi, functional magnetic resonance imaging; FA, fractional anisotropy; MD, mean diffusivity.

## Abbreviations:

ARBD: alcohol-related birth defects
ARND: alcohol-related neurodevelopmental disorders
BAC: blood alcohol concentration
DTI: diffusion tensor Imaging
FAS: fetal alcohol syndrome
FASD: fetal alcohol spectrum disorders
fMRI: functional magnetic resonance imaging
fPAT: functional photoacoustic tomography
GD: gestational day
MRI: magnetic resonance imaging
MRM: magnetic resonance microscopy
MRS: magnetic resonance spectroscopy
PAE: prenatal alcohol exposure
PAT: photoacoustic tomography
PET: positron emission tomography
pFAS: partial fetal alcohol syndrome
sPAT: structural photoacoustic tomography
SPECT: single-photon emission computed tomography
